# Quantification of artemisinin in human plasma using liquid chromatography coupled to tandem mass spectrometry

**DOI:** 10.1016/j.jpba.2008.12.014

**Published:** 2009-04-05

**Authors:** N. Lindegardh, J. Tarning, P.V. Toi, T.T. Hien, J. Farrar, P. Singhasivanon, N.J. White, M. Ashton, N.P.J. Day

**Affiliations:** a Faculty of Tropical Medicine, Mahidol University, Bangkok 10400, Thailand; b Nuffield Department of Clinical Medicine, Centre for Tropical Medicine, University of Oxford, Oxford, UK; c Hospital for Tropical Diseases, Ho Chi Minh City, Viet Nam; d Department of Pharmacology, Sahlgrenska Academy at University of Gothenburg, Gothenburg, Sweden

**Keywords:** Antimalarial, Artemisinin, High throughput, Liquid chromatography/tandem mass spectrometry (LC/MS/MS), Solid phase extraction (SPE)

## Abstract

A liquid chromatographic tandem mass spectroscopy method for the quantification of artemisinin in human heparinised plasma has been developed and validated. The method uses Oasis HLB™ μ-elution solid phase extraction 96-well plates to facilitate a high throughput of 192 samples a day. Artesunate (internal standard) in a plasma–water solution was added to plasma (50 μL) before solid phase extraction. Artemisinin and its internal standard artesunate were analysed by liquid chromatography and MS/MS detection on a Hypersil Gold C18 (100 mm × 2.1 mm, 5 μm) column using a mobile phase containing acetonitrile–ammonium acetate 10 mM pH 3.5 (50:50, v/v) at a flow rate of 0.5 mL/min. The method has been validated according to published FDA guidelines and showed excellent performance. The within-day, between-day and total precisions expressed as R.S.D., were lower than 8% at all tested quality control levels including the upper and lower limit of quantification. The limit of detection was 0.257 ng/mL for artemisinin and the calibration range was 1.03–762 ng/mL using 50 μL plasma. The method was free from matrix effects as demonstrated both graphically and quantitatively.

## 1 Introduction

Malaria is the most important parasitic disease of humans. An estimated 250 million people are infected annually with approximately one million deaths each year. The majority of infections and deaths occur in sub-Saharan Africa [1,2]. Multi-drug resistant *Plasmodium falciparum* malaria is today an increasing problem. Artemisinin (ARN) and its derivatives are still the most effective antimalarials available and artemisinin-based combination therapy (ACT) is recommended as first line treatment for *falciparum* malaria worldwide. Recent reports indicate that the artemisinins may be in jeopardy with increasing parasite clearance times and decreasing effectiveness on the Thai–Cambodian border [3–5]. ARN is a naturally occurring sesquiterpene lactone containing an endoperoxide group which is extracted from the Chinese herb *Artemisia annua*. ARN has very unusual pharmacokinetic properties with saturable first-pass metabolism and time-dependent pharmacokinetics upon repeated administration. Repeated oral administration over 5 days is associated with an increase in oral clearance, explained by an induction of metabolic capacity (i.e. autoinduction) [6–8]. Maximal ARN concentrations in healthy volunteers (*n* = 8) and in patients (*n* = 18) after a single oral dose of 500 mg ARN have been reported to be on average 450 and 587 ng/mL, respectively [9,10]. The rapid *in vivo* elimination of ARN in patients and the unusual clinical pharmacokinetic properties require a sensitive, robust and accurate bioanalytical method. Quantification of ARN and its derivatives has traditionally been problematic since the compounds do not have ultraviolet or fluorescent chromophores. Some analytical methods have reached a limit of detection of approximately 20–30 ng/mL by employing on-line post-column alkali derivatization before the UV detection [11–13]. Assays with electrochemical detection (ECD) in a reductive mode have also been developed utilizing the endoperoxide configuration of the compounds [14–16]. These methods have reached a limit of detection of 5–20 ng/mL using 0.5–1 mL plasma. The main drawback of ECD in reductive mode is that it requires rigorously controlled anaerobic conditions and deoxygenation of biologic samples as well as the mobile phase. This can be very difficult to establish and maintain practically. Two methods have been developed using chemiluminescent detection reaching a limit of detection of 17.5–25 ng/mL when using 0.5 and 1 mL of serum, respectively [17,18]. These two methods use post-column alkali derivatization and ultraviolet irradiation respectively to form the hydrogen peroxide used for chemiluminescent detection. The introduction of liquid chromatography mass spectrometry (LC–MS) and liquid chromatography tandem mass spectrometry (LC–MS/MS) during the last 10 years has revolutionized drug analysis and is today considered the gold standard in the analysis of drugs in biological fluids. To date, only two methods have been developed and validated for drug quantification of artemisinin in a biological matrix using LC–MS/MS [19,20]. The two methods focus on the quantification of artemisinin in rat serum or rat plasma and its application in pharmacokinetic studies. Sample pre-treatment consisted of liquid–liquid extraction or protein precipitation and the methods achieved limits of quantification of 1 and 4 ng/mL when using 100 μL of plasma or serum.

The aim of this work was to develop a sensitive and robust high throughput LC–MS/MS method using a low plasma volume (50 μL) to facilitate detailed human pharmacokinetic studies and therapeutic drug monitoring. The method has been validated according to published FDA guidelines [21,22].

## 2 Experimental section

### 2.1 Chemicals and materials

**Fig. 1:**
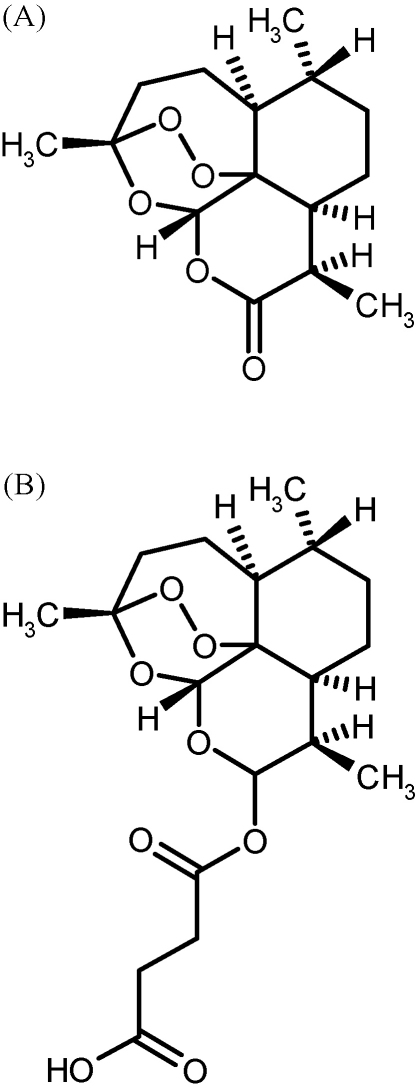
Structures of ARN (A) and ARS (B).

Artemisinin (ARN) and artesunate (ARS) were obtained from Guangzhou University of Traditional Chinese Medicine (Guangzhou, China). The structures are shown in Fig. 1. Acetonitrile (HPLC-grade), methanol (pro analysis) and HPLC-water were obtained from JT Baker (Phillipsburg, USA). Ammonium acetate (LC–MS grade) was obtained from FLUKA (Sigma–Aldrich, St. Louis, USA). Ammonium acetate buffer solutions were prepared by dissolving appropriate amounts of ammonium acetate in HPLC-water and adjusting pH with acetic acid (Merck Darmstadt, Germany).

### 2.2 Instrumentation—liquid chromatography mass spectrometry

The LC system was an Agilent 1200 system consisting of a binary LC pump, a vacuum degasser, a temperature-controlled micro well plate autosampler set at 4 °C and a thermostatted column compartment set at 40 °C (Agilent technologies, Santa Clara, USA). Data acquisition and quantification were performed using Analyst 1.4 (Applied Biosystems/MDS SCIEX, Foster City, USA). The compounds were analysed on a Hypersil Gold C18 (100 mm × 2.1 mm, 5 μm) column protected by a security guard column with a Hypersil Gold C18 (10 mm × 2.1 mm, 3 μm) guard cartridge (Thermo electron corporation, USA) under isocratic conditions using a mobile phase containing acetonitrile–ammonium acetate 10 mM pH 3.5 (50:50, v/v) at a flow rate of 500 μL/min with a wash out gradient. The complete LC gradient program is listed in Table 1. An API 5000 triple quadrupole mass spectrometer (Applied Biosystems/MDS SCIEX, Foster City, USA), with a TurboV™ ionisation source (TIS) interface operated in the positive ion mode, was used for the multiple reaction monitoring (MRM) LC–MS/MS analysis. The mass spectrometric conditions were optimized for the compounds by infusing a 100 ng/mL standard solution in mobile phase at 10 μL/min using a Harvard infusion pump (Harvard Apparatus, Holliston, USA) connected directly to the mass spectrometer. An additional tuning optimization of gas flows and temperatures was performed by continuously infusing the same standard solution at 10 μL/min via a “T” connector into the post-column mobile phase flow (500 μL/min). The TIS temperature was maintained at 475 °C and the TIS voltage was set at 4500 V. The curtain gas was set to 25.0 psi, and the nebulizer (GS1) and TIS (GS2) gases at 55.0 and 60.0 psi, respectively. The CAD gas in the collision cell was set at 5 psi. Quantification was performed using selected reaction monitoring (SRM) for the transitions *m*/*z* 300–209 for ARN and 402–267 for ARS. The declustering potential (DP) was 40.0 V for ARN and 50.0 V for ARS.

### 2.3 Preparation of plasma standards

Stock solutions of ARN and ARS (1000 μg/mL) were prepared in ethanol stored at −80 °C until use. Working solutions of ARN (ranging from 51.4 to 143,000 ng/mL) were prepared by serial dilution of the stock solution in ethanol–water (50–50, v/v). A 100 μL aliquot of each working solution (ARN) was added to blank heparin plasma (4900 μL) kept on ice to yield spiked calibration standards at six different concentrations ranging from 1.03 to 762 ng/mL. A calibration curve was constructed using 50 μL plasma of each standard. Quadratic regression with peak area ratio (drug/internal standard response) against concentration with 1/concentration^2^ (*x*^2^) weighting was used for quantification. Quality control (QC) samples for determination of accuracy and precision in heparin plasma at three concentrations (2.89, 40.7 and 571 ng/mL) were prepared in the same manner as the calibration standards and stored at −80 °C until analysis. The amount of stock solution in all spiked samples was kept at 2% of the total sample volume to minimize any systematic errors between real samples and standards. The calibration standards and QC samples were stored in cryovials at −80 °C until analysis. A working solution of ARS (5000 ng/mL) was prepared in ethanol and stored in 200 μL aliquots at −80 °C until use. On the day of assay this working solution was diluted with a plasma–water solution (50–50, v/v containing sodium fluoride/potassium oxalate 2/3 mg/mL) to 20.4 ng/mL and kept on ice until use.

### 2.4 Analytical procedure

An eppendorf stream multistepper was used to add 150 μL ice-cold internal standard solution (20.4 ng/mL ARS in plasma–water (50–50, v/v) containing sodium fluoride/potassium oxalate 2/3 mg/mL) to 50 μL plasma in a 96-well plate kept on ice. The samples were loaded onto a conditioned μ-elution HLB SPE 96-well plate (Waters, USA). All steps in the solid phase extraction (SPE) procedure were conducted using a 12-channel pipette as follows: the SPE wells were activated and conditioned using 750 μL acetonitrile followed by 750 μL methanol and 200 μL water. The samples (200 μL) were loaded onto the SPE plate and drawn through using a low vacuum. The SPE wells were washed using 300 μL water drawn through using medium vacuum. Full vacuum was applied briefly before the SPE column tips were wiped dry with tissue paper and a 96-collection plate (0.5 mL) was inserted into the vacuum manifold. The wells were eluted using 100 μL methanol–acetonitrile (90:10, v/v) drawn through using a low vacuum followed by 100 μL water. The SPE eluates were mixed briefly on a mixmate™ (Eppendorf, Germany) at 600 rpm and equilibrated at 4 °C overnight before analysis. 5 μL was injected into the LC–MS/MS system.

### 2.5 Validation

Linearity and the calibration model were evaluated using calibration curves obtained over 4 days. Precision and accuracy throughout the calibration range were evaluated by analysis of 5 replicates of quality control (QC) samples at three different concentrations daily for 4 days. Lower and upper limits of quantifications were evaluated by analysis of three replicates daily for 4 days. Carry-over effects for all the compounds were evaluated by injection of blank samples directly after injection of the highest point in the calibration curve. Over-curve dilution was evaluated by analysis of three replicates (2860 ng/mL ARN diluted five times with blank human heparin plasma) daily for 4 days. Stability of stock solutions in ethanol was evaluated at −80 °C. Stability of ARN in human heparin plasma was evaluated over three freeze/thaw cycles, at ambient temperature for 2 h and at 4 °C for 48 h. Bench-top stability of ARN in the autosampler was evaluated for 24 h. The concentrations were determined with 1/concentration^2^ weighted quadratic regression using a calibration curve prepared each day. Intra-, inter- and total-assay precisions were calculated using analysis of variance (ANOVA). Selectivity was evaluated by analysis of blank heparin plasma from six different donors. The potential interference between ARN and the internal standard ARS was also evaluated. Recovery was determined by comparing the peak area for extracted QC samples with direct injected solution containing the same nominal concentration of the compounds as the QC samples after SPE. Matrix effects were evaluated thoroughly using blank heparin plasma from six different donors. A quantitative estimation of the matrix effects was obtained by comparing the peak area for samples spiked in elution solution with extracted blank matrix spiked with the same nominal concentration of the compounds. A qualitative visualization of the matrix effects was obtained through post-column infusion experiments as described by others. Briefly, a continuous post-column infusion of 100/50 ng/mL ARN/ARS solution at 10 μL/min by a Harvard infusion pump through a T-connector was introduced to the mass spectrometer while samples to be tested were injected.

## 3 Results and discussion

**Fig. 2:**
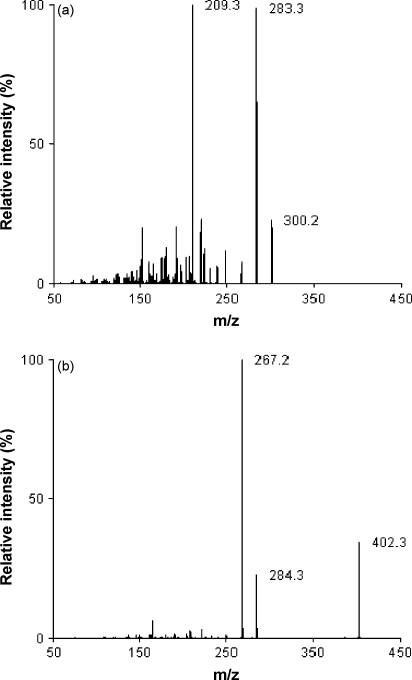
Collision-induced dissociation mass spectra for ARN (a) and ARS (b). For experimental conditions see Section 2.2.

Both electrospray (TIS) and atmospheric pressure chemical ionisation (APCI) have been used previously for quantification of artemisinin derivatives in biological fluids [19,23–28]. Previous investigations revealed that TIS was superior to APCI for the quantification of artesunate and dihydroartemisinin (DHA) mainly because of improved linearity [28]. The TIS interface provided good sensitivity for ARN and the mass spectra for both ARN and ARS displayed the base peak for the ammonium adduct (*m*/*z* 300) with significantly lower intensity for the protonated molecular ion [M+H]^+^ (*m*/*z* 283). The collision-induced dissociation (CID) mass spectra for ARN and ARS are shown in Fig. 2a and b. A mobile phase of acetonitrile–ammonium acetate 10 mM pH 3.5 (50:50, v/v) provided adequate retention and separation of ARN and the internal standard ARS. During method development and pre-validation ARS and DHA were evaluated as potential internal standards. The performance (i.e. with respect to intra- and inter-assay precision) was slightly better when using ARS compared to DHA. ARS eluted much closer (1.75 min) to ARN (2.06) with the current LC settings than DHA (1.38) making ARS more suitable to compensate for unforeseen matrix effects. Another advantage of ARS rather than DHA as the internal standard was the better stability [29]. The Oasis μ-elution HLB SPE 96-well plate facilitates low elution volumes and is designed for low sample volumes. Different elution solution compositions were evaluated to maximize recovery and facilitate direct injection from the eluate without causing band broadening of the peaks. A 100 μL of the elution solution (methanol–acetonitrile, 90–10, v/v) completely eluted the compounds from the SPE wells and the subsequent step using 100 μL of water was drawn through the SPE wells to dilute the organic solvent content and optimize compatibility with the LC–MS/MS system.

### 3.1 Validation

**Fig. 3:**
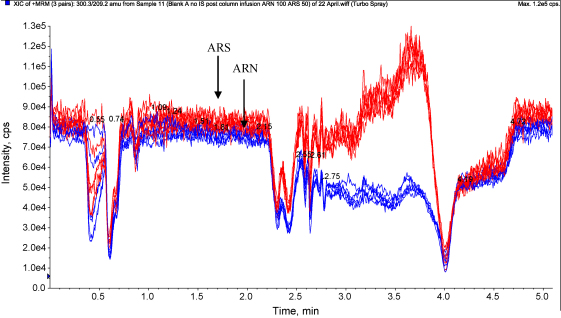
Overlays from injection of six extracted blank human plasma samples during post-column infusion 10 μL/min of ARN/ARS, 100/50 ng/mL.

The calibration range was determined to 1.03–762 ng/mL for ARN which includes the average maximal concentration after a single oral dose of 500 mg ARN in patients and healthy volunteers [9,10]. Back-calculated concentrations for the calibration standards were used to verify that the most appropriate regression model was used (Table 2). A quadratic regression model with 1/concentration^2^ (*x*^2^) weighting generated an evenly distributed low error over the whole calibration range with excellent reproducibility. Three different QC levels were evaluated over 4 consecutive days showing within-day, between-day and total-assay precisions (calculated using ANOVA single factor) below 6% for all three QC levels (Table 3). Limit of detection was determined to 0.257 ng/mL for artemisinin as it generated a peak that was clearly distinguishable from the background noise (i.e. >3 times the background variability seen in blank plasma). The LLOQ and ULOQ were set to 1.03 and 762 ng/mL, respectively, resulting in high precision and accuracy (Table 4). Neither ARN nor the internal standard produced any detectable carry-over after three injections of ULOQ. Plasma samples with concentrations above ULOQ could be diluted five times with blank plasma to fall within the calibration range without reduced accuracy or precision (Table 4). Blank human plasma from six different sources showed no interference with ARN. Interfering signals from blank plasma contributed less than 20% of the ARN signal at LLOQ. There was no interference of ARN on the internal standard or vice versa. The recovery (including matrix effects) was high (110–125%) at all tested concentrations for ARN and the internal standard. The reason for the recovery being higher than 100% is partly explained by a small enhancement of the signal and partly explained by the fact that a small fraction of the elution volume (150 μL) remains trapped on the SPE column. A small enhancement for ARN and the internal standard could be detected when references in neat injection solvent were compared with references in extracted blank biological matrix. The normalized matrix effects (ARN/ARS) were close to 1 with a low variation in accordance with international guidelines (Table 5) [22]. Post-column infusion experiments confirmed the absence of regions with severe matrix effects (i.e. no sharp drops or increases in the response) for blank human plasma extracted with the developed method (Fig. 3). ARN was found stable in heparinised plasma during three freeze/thaw cycles and for at least 2 h at ambient temperature or for up to 48 h when stored at 4 °C (i.e. >85% recovered). The stock solution was stable for at least 4 h at ambient temperature and for up to 14 days at −80 °C. ARN is stable for at least 4 h when prepared for extraction and for at least 24 h in the autosampler. The stability of the artemisinin derivates varies considerably in different sources of plasma and generally poor thermal stability is displayed [29,30]. The stability in plasma is significantly improved when stored on ice and the entire analytical process (including sample preparation for QC, standards and clinical samples) was therefore performed on ice [30].

### 3.2 Clinical applicability

**Fig. 4:**
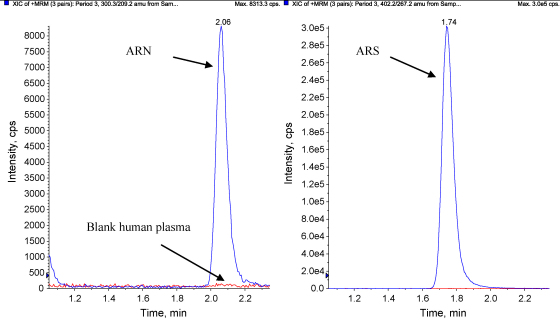
Chromatogram from a plasma sample (3.49 ng/mL ARN) taken 12 h after a single oral dose of 160 mg ARN from a healthy volunteer. Overlay of blank plasma.

**Fig. 5:**
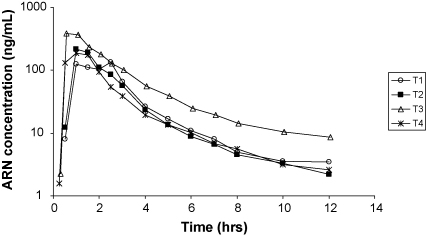
Concentration–time profiles from one healthy volunteer after four different treatments. T1: 160 mg ARN (new formulation), T2: 160 mg ARN (standard Vietnamese formulation), T3: 500 mg ARN (standard Vietnamese formulation), and T4: 160 mg ARN (standard Vietnamese formulation) coformulated with 720 mg piperaquine.

This assay was implemented for the analysis of clinical samples from a bioequivalence study in healthy volunteers in Vietnam. The analysed samples were from a single-centre, cross-over study in 15 healthy subjects to determine the relative bioavailability of artemisinin following oral administration of four different formulations, each dose occasion separated by a washout period of 3 weeks. The four arms were; single oral administration of 160 mg ARN (new formulation), 160 mg ARN (standard Vietnamese formulation), 500 mg ARN (standard Vietnamese formulation), and 160 mg ARN (new formulation) coformulated with 720 mg piperaquine. The full results will be described in full elsewhere. Plasma samples were collected for up to 12 h after dose and Fig. 4 shows a chromatogram from a healthy volunteer sample taken 12 h after an oral dose of ARN (160 mg, new formulation). The chromatogram displays an ARN concentration of 3.49 ng/mL with an overlay of a blank plasma sample. None of the measured ARN concentrations at 12 h after dose fell below the LLOQ and only one sample out of the 900 analysed was above the ULOQ displaying excellent clinical applicability for the developed method. The sample above the ULOQ was successfully diluted to fall within the calibration range. Fig. 5 displays concentration–time profiles for the different treatment arms for one healthy volunteer.

## 4 Conclusion

A sensitive high throughput LC–MS/MS method for the determination of ARN in human heparin plasma has been developed and validated. The assay presented here is the first validated method to quantify ARN in human plasma using LC–MS/MS. The total analysis time for two batches (192 samples) is less than 24 h. The assay had a high recovery and proved to be highly sensitive with excellent reproducibility. This method requires only 50 μL of plasma and ARN is stable in human plasma for up to 2 h at ambient temperature but will remain stable for considerably longer if kept on ice. The developed method is shown to be ideal for pharmacokinetic studies and will hopefully provide increased knowledge about the clinical features of artemisinin as stability, sensitivity and accuracy has been improved compared to previously published assays.

## Figures and Tables

**Table 1:** LC gradient programme.

Time (min)	% solvent A	% solvent B	Flow (μL/min)
0	100	0	500
0.8	100	0	500
1.0	0	100	500
2.2	0	100	500
2.3	0	100	1000
3.0	0	100	1000
3.1	100	0	1000
4.6	100	0	750
4.7	100	0	500
5.1	100	0	500

Solvent A: acetonitrile–aqueous ammonium acetate 10 mM pH 3.5 (50–50, v/v). Solvent B: methanol–acetonitrile (75–25, v/v).

**Table 2:** Back-calculated concentrations of standard curves for artemisinin in human plasma.

Nominal concentration (ng/mL)	1.03	3.85	14.4	54.2	203	762
Average (*n* = 8)	1.02	3.88	14.6	54	201	765
S.D.	0.01	0.15	0.35	3.21	5.6	19.6
CV (%)	1.28	3.95	2.39	5.99	2.78	2.56
Accuracy	99.6	101	101	98.7	99.2	100

**Table 3:** Inter-, intra- and total-assay precision (ANOVA) for artemisinin in human plasma.

	Inter-assay (%)	Intra-assay (%)	Total-assay (%)
QC 1: 2.89 ng/mL	5.46	3.98	4.25
QC 2: 40.7 ng/mL	3.24	2.62	2.72
QC 3: 571 ng/mL	4.35	3.32	3.51

**Table 4:** Inter-, intra- and total-assay precision (ANOVA) for lower limit of quantification, upper limit of quantification and diluted over-curve samples for artemisinin in human plasma.

	Inter-assay CV (%)	Intra-assay CV (%)	Total-assay CV (%)	Accuracy^a^ (%)
LLOQ: 1.03 ng/mL	2.82	3.53	3.35	101
ULOQ: 762 ng/mL	8.02	2.16	4.57	99
Over-curve: diluted to 572 ng/mL	3.56	3.57	3.57	104

a Mean accuracy over all 4 validation days.

**Table 5:** Matrix effects. Artemisinin (ARN) and internal standard (ARS) spiked in extracted blank human plasma *vs.* spiked in elution solution.

	Blank A	Blank B	Blank C	Blank D	Blank E	Blank F	Average	S.D.	CV (%)
ARN (QC1): 2.89 ng/mL	105	107	110	106	101	108	106	3.0	2.9
ARS (QC1): 20.4 ng/mL	109	110	106	108	109	116	110	3.4	3.1
Normalized response	0.96	0.98	1.04	0.99	0.93	0.93	0.97	0.04	4.1

ARN (QC3): 571 ng/mL	102	108	106	107	106	108	106	2.2	2.1
ARS (QC3): 20.4 ng/mL	109	107	112	115	112	116	112	3.4	3.0
Normalized response	0.93	1.01	0.94	0.92	0.95	0.93	0.95	0.03	3.3
